# Elderly patients with atrial fibrillation in routine clinical practice—peri-procedural management of edoxaban oral anticoagulation therapy is associated with a low risk of bleeding and thromboembolic complications: a subset analysis of the prospective, observational, multinational EMIT-AF study

**DOI:** 10.1186/s12872-020-01766-w

**Published:** 2020-12-01

**Authors:** M. Unverdorben, C. von Heymann, A. Santamaria, M. Saxena, T. Vanassche, J. Jin, P. Laeis, R. Wilkins, C. Chen, P. Colonna

**Affiliations:** 1 Global Medical Affairs Specialty and Value Products, Daiichi Sankyo Inc., 211 Mt Airy Road, Basking Ridge, NJ 07920 USA; 2 Department of Anaesthesia and Intensive Care Medicine, Emergency Medicine, and Pain Therapy, Vivantes Klinikum Im Friedrichshain, Landsberger Allee 49, 10249 Berlin, Germany; 3 Hematology Department, University Hospital Vilaopó y Torrevieja, Alicante, Spain; 4 William Harvey Research Institute, Barts Health NHS Trust, Charterhouse Square, London, EC1M 6BQ UK; 5 Department of Cardiovascular Sciences, University Hospitals (UZ) Leuven, Leuven, Belgium; 6 Daiichi Sankyo, Medical Affairs Europe, Munich, Germany; 7 QPS Consulting, LLC, 19884 Naples Lakes Terrace, Ashburn, VA 20147 USA; 8 Department of Cardiology, Polyclinic of Bari - Hospital, 70124 Bari, Italy

**Keywords:** Age, Bleeding, Systemic thromboembolism, Atrial fibrillation, Edoxaban

## Abstract

**Background:**

Annually > 10% of patients with atrial fibrillation on oral anticoagulation undergo invasive procedures. Optimal peri-procedural management of anticoagulation, as judged by major bleeding and thromboembolic events, especially in the elderly, is still debated.

**Methods:**

Procedures from 1442 patients were evaluated. Peri-procedural edoxaban management was guided only by the experience of the attending physician. The primary safety outcome was the rate of major bleeding. Secondary outcomes included the peri-procedural administration of edoxaban, other bleeding events, and the main efficacy outcome, a composite of acute coronary syndrome, non-hemorrhagic stroke, transient ischemic attack, systemic embolic events, deep vein thrombosis, pulmonary embolism, and mortality.

**Results:**

Of the 1442 patients, 280 (19%) were < 65, 550 (38%) were 65–74, 514 (36%) 75–84, and 98 (7%) were 85 years old or older. With increasing age, comorbidities and risk scores were higher. Any bleeding complications were uncommon across all ages, ranging from 3.9% in patients < 65 to 4.1% in those 85 years or older; major bleeding rates in any age group were ≤ 0.6%. Interruption rates and duration increased with advancing age. Thromboembolic events were more common in the elderly, with all nine events occurring in those > 65, and seven in patients aged > 75 years.

**Conclusion:**

Despite increased bleeding risk factors in the elderly, bleeding rates were small and similar across all age groups. However, there was a trend toward more thromboembolic complications with advancing age. Further efforts to identify the optimal management to reduce ischemic complications are needed.

*Trial registration*: NCT# 02950168, October 31, 2016

## Background

Aging is associated with a decline of many physiologic functions, determined by factors that include genetic predisposition, environmental influences, and lifestyle choices. However, there is considerable variation in the impact of aging on individual responses to disease [1]. In cardiovascular medicine, the number and severity of risk factors increases in the elderly, leading to a higher incidence of diseases such as atrial fibrillation (AF), along with sequelae such as systemic thromboembolism and stroke [2]. The interaction of the body with therapeutic measures changes too, as reflected by altered pharmacokinetics and pharmacodynamics [3], associated with changes in glomerular filtration rate, decreased protein binding and decrease in liver function. Therefore, in therapies that cover a wide range of ages, it is prudent to investigate the benefits and risks stratified by age groups [4].

Based on US data from 2002 [5], an adult undergoes an average of approximately nine surgical procedures in a lifetime, with a rate that increases with age, reaching a rate of 0.16 operations/person/year at age 75. With over 33 million patients with AF (2010 data) globally [6], there is a large population at need of oral anticoagulation in atrial fibrillation to reduce the risk of thromboembolic events.

An estimated 10% of patients treated with oral anticoagulants are annually subjected to diagnostic or therapeutic procedures [7] of varying invasiveness and, thus, to the risk of severe bleeding and ischemic events [8]. All procedures with a bleeding risk require optimization of the peri-procedural management of anticoagulation: whether to interrupt oral anticoagulation at all and if so, for how long and when, relative to the procedure. As patients of advanced age, especially the very elderly aged 85 years and above [9] are often undertreated with oral anticoagulants because of concerns with regard to increased bleeding risk, and lack of benefit in stroke prevention [10, 11]. It is valuable to understand whether older patients are also treated differently peri-procedurally from younger patients.

The EMIT (Edoxaban Management in Diagnostic and Therapeutic Procedures) study [12] prospectively investigated the peri-procedural management and outcome in a large cohort of unselected patients who were treated with the direct oral anticoagulant edoxaban for any of its approved indications. Adding to the EMIT results, this paper discusses the safety and efficacy of edoxaban in elderly patients from those aged ≥ 65 years of age to the very elderly, aged 85 years. Although the majority of patients with AF are elderly, and so typical patient management might be assumed to meet the needs of the elderly, a detailed cohort analysis allows understanding of when, as aging progresses, differences in risk and tactics for treatment and risk mitigation become identifiable.

## Methods

### Design

The EMIT study was a multicentre, prospective, and noninterventional study integrating data from seven European and four Asian countries. The detailed design has been published [13, 14]. The study was conducted in accordance with the Declaration of Helsinki and with local Institutional Review Board approvals and was registered as NCT02950168. Written informed consent was obtained from participants prior to enrolment. The peri-procedural management of anticoagulant therapy was at the discretion of the investigator, including any decision whether to interrupt edoxaban therapy and the timing/duration of any interruption. No attempt was made to influence patient management by the study authors, study team or the sponsor.

### Objectives and outcome parameters

The objective of the registry was to analyse the peri-procedural management of patients receiving edoxaban in daily practice and to collect data on safety and other outcome parameters in these patients. The primary safety outcome was the rate of major bleeding (MB) from five days prior to the procedure to 30 days post-procedure using the International Society of Thrombosis and Haemostasis (ISTH) definition [15].

Secondary outcomes included exploration of the main efficacy outcome, defined as the composite of acute coronary syndrome (ACS), stroke, transient ischemic attack (TIA), systemic embolic events (SEE), deep vein thrombosis (DVT), pulmonary embolism (PE), and cardiovascular (CV) mortality, along with the individual components. Other secondary outcomes included the incidences of clinically relevant non-major bleeding (CRNMB) and all-cause mortality. CRNMB events were defined as overt bleeding that required medical attention but that did not fulfil the criteria for MB. Other secondary outcomes of the study were evaluation of the peri-procedural interruption and dosing of edoxaban. All incidents of MB, CRNMB, ACS, and acute thromboembolic events were reviewed and unanimously adjudicated by the Steering Committee. Bleeding had to commence during or after the procedure to be classified as a procedural complication.

### Patient recruitment

Enrolment commenced in December 2016. Subjects were recruited consecutively. Eligible patients were ≥ 18 years of age, had AF, were treated with edoxaban according to the local labels, were not enrolled in any other study concurrently, and underwent any type of diagnostic or therapeutic procedure. Data from the first procedure in each patient is reported.

### Observations

The observation period of the study started five days before the procedure and ended 30 days afterward. Date of visit, details of edoxaban treatment, and clinical outcomes were documented at 30 days after each procedure. No interruption of edoxaban therapy was defined if edoxaban was administered on each day of the observation period. Any interruption of edoxaban treatment was recorded as the number of days without administration of edoxaban, pre-procedural and/or post-procedural. Any dose skipped before or on the day of the procedure was defined as pre-procedural.

Peri-procedural EHRA bleeding risk [16]; HAS-BLED [17] (Hypertension, Abnormal renal/liver function, Stroke, Bleeding history or predisposition, Labile international normalized ratio, Elderly, Drugs/alcohol concomitantly) score; CHA_2_DS_2_-VASc (Congestive heart failure, Hypertension, Age ≥ 75 [doubled], Diabetes, Stroke [doubled]-Vascular disease, Age 65–74 years, and gender [female]) score [18]; details of edoxaban treatment; diagnostic/therapeutic procedures; and clinical findings were documented at baseline and during the peri-procedural period.

### Statistical analysis

Binary, categorical, and ordinal parameters were summarized by means of absolute and percentage numbers. Numerical data were described by standard statistics. The statistical analyses were performed using SAS^®^ version 9.3 or higher (SAS Institute, Cary, North Carolina, USA).

In order to maximize the utility of the data, three sets of age cut-offs were utilized: less than 65 years versus 65 years and older; less than 75 years versus 75 years and older; a more granular set of four groups with < 65, ≥ 65 to < 75, ≥ 75 to < 85, and ≥ 85 years following UN standards [19]; and in line with the FDA [20], a second cut-off of 75 years and above was chosen. These multiple cut-offs permit analysis following both conventional definitions of the elderly and very elderly, as well as acknowledging that many populations are reaching advanced age in a healthier state than previous generations [21]. Use of multiple narrow cohorts, plus their larger combinations, permits analysis whatever definition of the elderly is chosen.

## Results

**Fig. 1:**
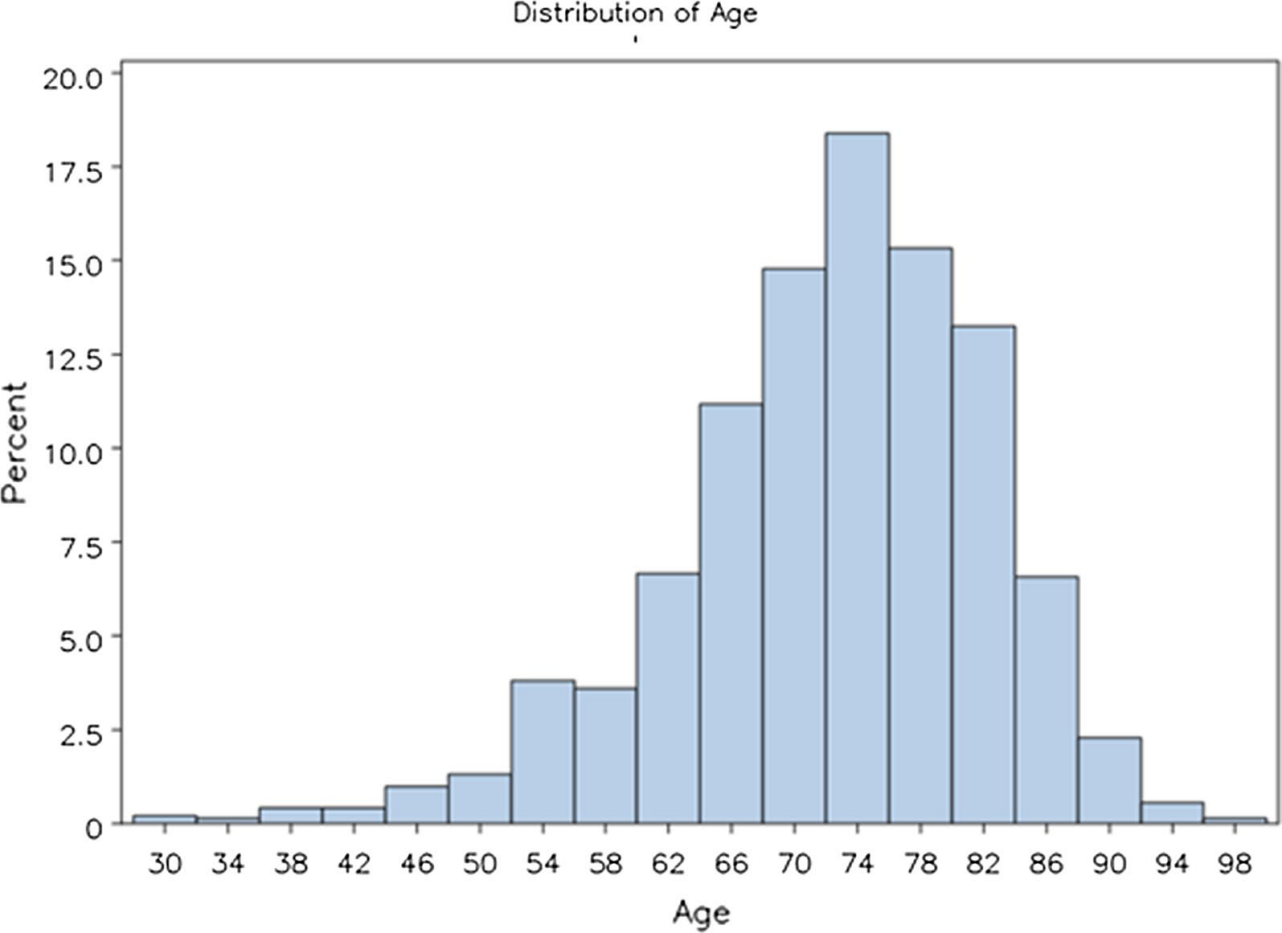
Age distribution of study population

The dataset included 1,442 patients’ first procedures. Of these patients, 280 (19%) were younger than 65 years, 550 (38%) were 65–74 years old, 514 (36%) 75–84 years old, and 98 (7%) were 85 years old or older. See Fig. 1.

In all age groups except those ≥ 85 years, the patients were predominantly male with the male proportion declining with age. All recorded comorbidities were more prevalent with increasing age, except obesity/overweight (assessed as BMI [22]) and dyslipidaemia, which trended lower in patients aged 85 years and older. A creatinine clearance of  ≤ 50 mL/min was found in 21% of patients aged from 75 to 85 years, and 49% of patients of 85 years or older. Use of the 30 mg edoxaban dose increased with age, being utilized in 9% of patients < 65 years, and in 68% of patients ≥ 85 years. Heparin bridging was used in about 10% of patients, with a slight trend toward greater use in the more elderly. HAS-BLED and CHA_2_DS_2_-VASc scores also increased with age, particularly in the over 75 age groups, (although it should be noted that age is a component of each score). Clinical characteristics and patient baseline data summarized in Table 1 were included as predictors of the composite event of major or CRNM bleeding, all cause mortality, stroke, SEE, ACS, VTE, and TIA in a logistic regression analysis. Due to likely correlations among predictors, a stepwise approach is applied. A significance level of 0.1 was set to allow a variable into the model and for a variable to stay in the model. The final selected model includes predictors of weight (p = 0.003), prior coronary heart disease (p = 0.029), prior use of heparin (p = 0.043), and prior use of P-gp inhibitors (p = 0.054). Weight, prior coronary heart disease, and prior use of heparin emerged as independent predictors. Low body weight, prior coronary heart disease, and prior heparin use are associated with higher event probability. Importantly, age was not an identified predictor of adverse outcomes.

**Table 1:** Patient background information

Age group (years)	< 65	≥ 65	< 75	≥ 75	≥ 65 to < 75	≥ 75 to < 85	≥ 85
# of subjects	280	1162	830	612	550	514	98
Age mean (SD)	56.5 (7.2)	75.5 (6.4)	65.4 (8.0)	80.5 (4.2)	69.9 (2.9)	79.1 (2.8)	87.8 (2.8)
Male n (%)	206 (73.6)	715 (61.5)	593 (71.4)	328 (53.6)	387 (70.4)	281 (54.7)	47 (48.0)
BMI mean (SD)	28.8 (5.4)	27.1 (4.7)	28.2 (5.1)	26.5 (4.5)	27.8 (4.8)	26.7 (4.6)	25.6 (3.9)
Hypertension n (%)	161 (57.5)	893 (76.9)	568 (68.4)	486 (79.4)	407 (74.0)	405 (78.8)	81 (82.7)
Dyslipidaemia n (%)	74 (26.4)	535 (46.0)	333 (40.1)	276 (45.1)	259 (47.1)	241 (46.9)	35 (35.7)
Diabetes mellitus n (%)	51 (18.2)	304 (26.2)	188 (22.7)	167 (27.3)	137 (24.9)	140 (27.2)	27 (27.6)
Coronary heart disease n (%)	30 (10.7)	272 (23.4)	144 (17.3)	158 (25.8)	114 (20.7)	135 (26.3)	23 (23.5)
Valvular heart disease n (%)	33 (11.8)	229 (19.7)	131 (15.8)	131 (21.4)	98 (17.8)	110 (21.4)	21 (21.4)
CrCl mean (SD)	104.5 (37.1)	67.0 (24.4)	86.2 (31.6)	57.3 (19.7)	77.8 (24.5)	59.7 (19.2)	44.7 (17.9)
CrCL ≤ 50 mL/min n (%)	1 (0.4)	179 (15.4)	25 (3.0)	155 (25.3)	24 (4.4)	107 (20.8)	48 (49.0)
CHA_2_DS_2_-VASc score mean (SD)	1.5 (1.1)	3.5 (1.4)	2.4 (1.3)	4.2 (1.3)	2.8 (1.2)	4.2 (1.3)	4.4 (1.2)
HAS-BLED score mean (SD)	0.9 (0.9)	2.0 (1.0)	1.6 (1.0)	2.1 (1.0)	1.9 (0.9)	2.1 (1.0)	2.1 (1.0)
EHRA bleeding risk-minor n (%)	36 (12.9)	263 (22.6)	147 (17.7)	152 (24.8)	111 (20.2)	124 (24.1)	28 (28.6)
EHRA bleeding risk-low n (%)	174 (62.1)	580 (49.9)	457 (55.1)	297 (48.5)	283 (36.4)	252 (39.1)	45 (45.9)
EHRA bleeding risk-high n (%)	62 (22.1)	289 (24.9)	199 (24.0)	152 (24.8)	137 (24.9)	129 (25.1)	23 (23.5)
EHRA risk unknown n (%)	8 (2.9)	30 (2.6)	27 (3.3)	11 (1.8)	19 (3.5)	9 (1.7)	2 (2.0)
30 mg daily edoxaban dose n (%)	25 (8.9)	341 (29.3)	126 (15.2)	240 (39.2)	101 (18.4)	173 (33.7)	67 (68.4)
Heparin “bridging” n (%)	134 (9.3)	113 (9.7)	71 (8.6)	(10.3)	50 (9.1)	51 (9.9)	12 (12.2)

All data presented as mean ± SD; number (%); or number, mean ± SD

See text for definition of HAS-BLED and CHA_2_DS_2_-VASc scores

*EHRA* European Heart Rhythm Association, *BMI* body mass index

The demographic and medical backgrounds of the evaluated patients are summarized in Table 1.

EHRA procedural bleeding risk distribution was similar across all age groups, although the proportion of minor risk procedures increased in older age groups.

In all patient age groups, vascular access and transcatheter diagnostics or interventions were the most frequently performed procedures. The next three most common procedures across all age groups were cardiovascular/vascular, gastroenterological, and dentistry procedures. Orthopaedic and ophthalmologic procedures were more common in those aged 75 years or more. See Table 2 for further details. Of 1,442 first procedures, 1,324 (92%) were pre-scheduled.

**Table 2:** Procedure groups by age range

Age (years) Procedure type	≤ 65	≥ 65	≤ 75	≥ 75	≥ 65 to < 75	≥ 75 to < 85	≥ 85	Total
N	280	1162	830	612	550	514	98	1442
Cardiothoracic and vascular surgery	27 (9.6%)	160 (13.8%)	85 (10.2%)	102 (16.7%)	58 (10.6%)	80 (15.6%)	22 (22.5%)	187 (13.0%)
Dentistry	22 (7.9%)	121 (10.4%)	79 (9.5%)	64 (10.5%)	57 (10.4%)	51 (9.9%)	13 (13.3%)	143 (9.9%)
Dermatology	4 (1.4%)	44 (3.8%)	21 (2.5%)	27 (4.4%)	17 (3.1%)	19 (3.7%)	8 (8.2%)	48 (3.3%)
Ear, nose, throat	1 (0.4%)	14 (1.2%)	10 (1.2%)	5 (0.8%)	9 (1.6%)	4 (0.8%)	1 (1.1%)	15 (1.0%)
Gastroenterology	24 (8.6%)	150 (12.9%)	105 (12.7%)	69 (11.3%)	81 (14.7%)	60 (11.7%)	9 (9.2%)	174 (12.1%)
Gynaecology	3 (1.1%)	10 (0.9%)	6 (0.7%)	7 (1.1%)	3 (0.6%)	5 (1.0%)	2 (2.0%)	13 (0.9%)
Haematology	0	2 (0.2%)	1 (0.1%)	1 (0.2%)	1 (0.2%)	1 (0.2%)	0	2 (0.2%)
Miscellaneous	5 (1.8%)	8 (0.7%)	11 (1.3%)	2 (0.3%)	6 (1.1%)	1 (0.2%)	1 (1.0%)	13 (0.9%)
Ophthalmology	5 (1.8%)	70 (6.0%)	27 (3.3%)	48 (7.8%)	22 (4.0%)	43 (8.4%)	5 (5.1%)	75 (5.2%)
Orthopaedic	7 (2.5%)	87 (7.5%)	38 (4.6%)	56 (9.1%)	31 (5.6%)	46 (9.0%)	10 (10.2%)	94 (6.5%)
Surgery (general)	12 (4.3%)	59 (5.1%)	40 (4.8%)	31 (5.1%)	28 (5.1%)	26 (5.0%)	5 (5.1%)	71 (4.9%)
Urology	3 (1.1%)	58 (5.0%)	32 (3.9%)	29 (4.7%)	29 (5.3%)	25 (4.9%)	4 (4.1%)	61 (4.2%)
Vascular access and transcatheter diagnostics and interventions	167 (59.6%)	379 (32.6%)	375 (45.2%)	171 (27.9%)	208 (37.8%)	153 (29.8%)	18 (18.4%)	546 (37.9%)

Edoxaban was discontinued pre-procedurally in 48% of all patients. Interruption rates were higher and duration of interruption longer in the older age groups. See Table 3 for details. Most patients aged 65 years or more had a post-procedural therapy interruption. Interruptions of two or more days were more common in patients aged 65 years or older (p < 0.001). Figure 3 shows the number of patients in each age group taking edoxaban on each peri-procedural day.

**Table 3:** Interruption characteristics by age group

Age (years)	N	No interruption	1 day interruption	≥ 2 days interruption
*Pre-procedural interuption duration*				
< 65	279	145 (52.0%)	54 (19.4%)	80 (28.7%)
≥ 65	1161	422 (36.3%)	320 (27.6%)	419 (36.1%)
< 75	828	365 (44.1%)	206 (24.9%)	257 (31.0%)
≥ 75	612	202 (33.0%)	168 (27.5%)	242 (39.5%)
≥ 65– < 75	549	220 (40.1%)	152 (27.7%)	177 (32.2%)
≥ 75– < 85	514	178 (34.6%)	138 (26.8%)	198 (38.5%)
≥ 85	98	24 (24.5%)	30 (30.6%)	44 (44.9%)
*Post-procedural interuption duration*				
< 65	279	226 (81.0%)	19 (6.8%)	34 (12.2%)
≥ 65	1157	810 (70.0%)	93 (8.0%)	254 (22.0%)
< 75	825	621 (75.3%)	56 (6.8%)	148 (17.9%)
≥ 75	611	415 (67.9%)	56 (9.2%)	140 (22.9%)
≥ 65– < 75	546	395 (72.3%)	37 (6.8%)	114 (20.9%)
≥ 75– < 85	514	351 (68.3%)	50 (9.7%)	113 (22.0%)
≥ 85	97	64 (66.0%)	6 (6.2%)	27 (27.8%)
*Interuption incidence*				
< 65	279	134 (48.0%)	92 (33.0%)	11 (3.9%)
65–74	549	194 (35.3%)	201 (36.6%)	26 (4.7%)
75–84	514	158 (30.7%)	193 (37.5%)	20 (3.9%)
≥ 85	98	21 (21.4%)	43 (43.9%)	3 (3.1%)
≥ 65	1161	373 (32.1%)	437 (37.6%)	49 (4.2%)
< 75	828	328 (39.6%)	293 (35.4%)	37 (4.5%)
≥ 75	612	179 (29.2%)	236 (38.6%)	23 (3.8%)

**Fig. 2:**
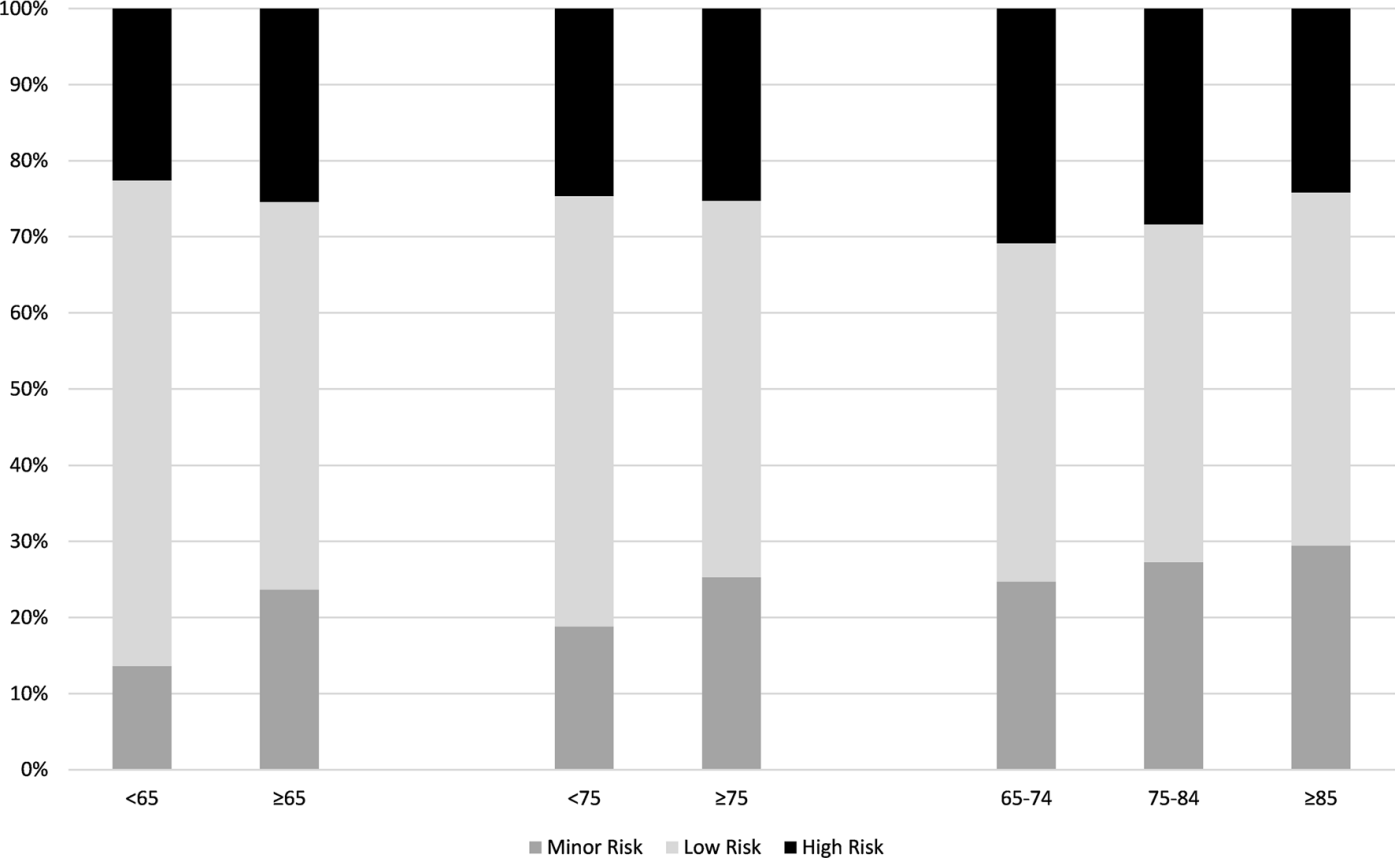
EHRA procedural bleeding risk (%) by age group. Chart excludes missing data (< 4% of records in any age group)

Bleeding occurred in 3.9% of patients < 65 years and 4.1% in those 85 years or older. Major bleeding and CRNMB had an overall incidence of 1.0%, with the highest incidence in patients aged 65–74 years (1.6%). There was no discernible relationship between age and all bleeding complications. However, ischemic/thromboembolic complications were more common in the elderly, with all of nine events occurring in those over 65 years, and seven (77.8%) in those patients of 75 years and older. See Table 4 for details.

**Table 4:** Clinical outcomes by age group

Outcome	Age < 65 years	Age ≥ 65 years	Age < 75 years	Age ≥ 75 years	Age ≥ 65– < 75 years	Age ≥ 75– < 85 years	Age ≥ 85 years	Total
Number of subjects	280	1162	830	612	550	514	98	1442
All bleeding	11 (3.9%)	39 (3.4%)	29 (3.5%)	21 (3.4%)	18 (3.3%)	17 (3.3%)	4 (4.1%)	50 (3.5%)
MB	1 (0.4%)	5 (0.4%)	3 (0.4%)	3 (0.5%)	2 (0.4%)	3 (0.6%)	0	6 (0.4%)
MB or CRNMB	2 (0.7%)	13 (1.1%)	11 (1.3%)	4 (0.7%)	9 (1.6%)	4 (0.8%)	0	15 (1.0%)
ACS	0	1 (0.1%)	1 (0.1%)	0	1 (0.2%)	0	0	1 (0.1%)
Stroke	0	5 (0.4%)	1 (0.1%)	4 (0.7%)	1 (0.2%)	3 (0.6%)	1 (1.0%)	5 (0.3%)
TIA	0	1 (0.1%)	0	1 (0.2%)	0	1 (0.2%)	0	1 (0.1%)
VTE	0	1 (0.1%)	0	1 (0.2%)	0	1 (0.2%)	0	1 (0.1%)
SEE	0	1 (0.1%)	0	1 (0.2%)	0	1 (0.2%)	0	1 (0.1%)
CV mortality	0	3 (0.3%)	1 (0.1%)	2 (0.3%)	1 (0.2%)	2 (0.4%)	0	3 (0.2%)
All-cause mortality	0	9 (0.8%)	6 (0.7%)	3 (0.5%)	6 (1.1%)	2 (0.4%)	1 (1.0%)	9 (0.6%)

## Discussion

We report the impact of age on the management and incidence of critical thromboembolic and bleeding events in patients with AF treated with the direct oral anticoagulant edoxaban. While bleeding rates were low, thromboembolic rates were higher in the elderly. This study complements the PAUSE and DRESDEN studies [8, 23], by describing outcomes associated with edoxaban administration, and describing use in a routine clinical practice setting, and provides additional data on the impact of age on outcome.

Data was collected, in the first prospective observational multicentre multinational study on the management and outcomes of edoxaban therapy, on 1442 unselected procedures in AF patients. All common comorbidities increased with age, except for obesity and dyslipidaemia, which were lower in patients 85 years and older. Of those patients aged from 75 to 85 years, 21% had a creatinine clearance of ≤ 50 mL/min; 49% of patients 85 years or older had a creatinine clearance of ≤ 50 mL/min. Use of 30 mg edoxaban dose was more common in the older age groups, being taken by 9% of patients < 65 years, and 68% of patients at least 85 years of age. HAS-BLED and CHA_2_DS_2_-VASc scores also increased with age (although of note age is a component of each score). Overall, and as expected [11, 24], in older patients the ischemic cardiovascular risk was more clinically significant than the bleeding risk. Despite detailed analysis (Table 1) across multiple age cohort groups, no other factors linked to age were identified. This data supports the case that, if dose is appropriately adjusted based upon label requirements, elderly patients do not require modification of management.

Edoxaban interruption rates and duration of interruption increased with age. The difference in post-procedural interruption across age groups (> 2-day post-procedural interruption 12.2% in patients < 65 vs. 27.8% in patients ≥ 85 years), suggests physicians tend to be cautious in resuming edoxaban in older patients.

Any bleeding complications were rare across all ages. Major bleeding (0.4%), and CRNMB had a combined incidence of 1.0%, with the highest incidence in patients aged 65–74 years (1.6%). There was no discernible relationship between age and bleeding complications. Ischemic or thromboembolic complications were, however, more common in the elderly.

Patients aged 65 years and above had combined rates of MB and CRNMB only slightly higher than those < 65 years (1.1% vs. 0.7%) but more ischemic/thromboembolic events (0.8% vs. 0%). Possible explanations include procedures with a higher EHRA bleeding risk being performed in the advanced age groups; or differences in the interruption of edoxaban; or age as an independent risk factor per se. EHRA risk category is an unlikely explanation in patients of 65 years and above as this factor is only slightly more prevalent than in younger individuals: indeed EHRA minor risk procedures were more common in the elderly (see Fig. 2). A more likely explanation is the duration of peri-procedural interruption of edoxaban therapy, which shows a trend toward increased duration with age and appears to be independent from the EHRA bleeding risk. Longer interruption of edoxaban therapy with the intent to reduce bleeding appears to come at the cost of increased risk of thromboembolic/ischemic events. The increase in thromboembolic rates across the age groups was from zero events under age 65 to nine events in the 65-and-older group, while in the same groups the MB/CRNMB rate differed by only 0.4% (0.7% to 1.1%). Prolonged interruption of therapy in the elderly is not associated with a lower risk of major bleeding, but appears to be associated with a higher thromboembolic risk. Other studies have evaluated peri-procedural risk [8, 23, 25] but have not provided results stratified by age. The PAUSE study [8], which included 3007 procedures, reported post-procedural rates of MB of 1.35% for apixaban, 0.90% for dabigatran, and 1.85% for rivaroxaban cohorts with arterial thromboembolism occurring in 0.16%, 0.6%, and 0.37%, respectively. RE-LY [25] reported a thromboembolism rate of 0.35% and a MB rate of 1.62% in patients treated with dabigatran. The DRESDEN Registry [23] reported peri-procedural MB rates of 1.2% and cardiovascular event rates of 1% in a series of 863 procedures in patients who were mainly treated with rivaroxaban. Although different study designs, the event rates in EMIT and all these studies are similar. The four studies together indicate that oral anticoagulation when managed carefully is associated with a low incidence of major haemorrhagic and/or significant thromboembolic events.

**Fig. 3:**
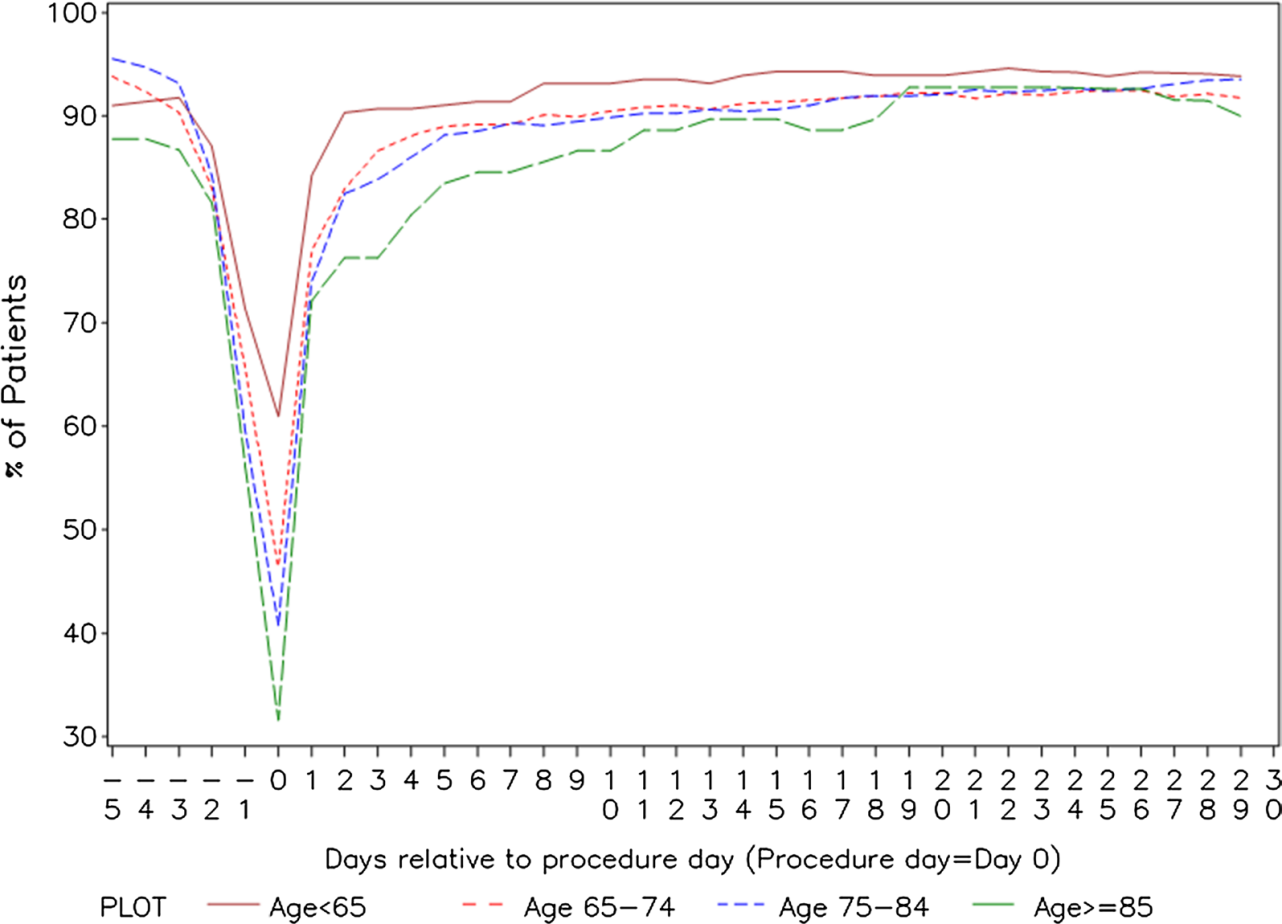
Time course of edoxaban interruption

Several studies have evaluated DOAC use in the elderly; however, many have not collected peri-procedural data. Halperin et al. [26], reporting the ROCKET-AF comparison of rivaroxaban with VKA in patients with AF, noted increased thromboembolic (4.61 events/100 patient-years vs. 6.07) and MB rates (2.69 vs. 4.86) in the patients aged < 75 or ≥ 75 years, respectively. The RE-LY study [27] evaluated patients randomized to dabigatran or warfarin. Thromboembolic rates (per patient-year) increased from 1.32% in subjects < 65 years of age, to 1.95% in those aged 80—84 years. The MB rate was 0.14% in subjects < 65 years of age and 5.01% in those aged 80—84 years. In patients with AF not undergoing procedures, the risk of stroke increases 1.5-fold for each decade [28].

### Limitations of study

One limitation of this study is a lack of a comparator arm and its restriction to one DOAC, although this study provides data on peri-procedural management of edoxaban for the first time. The much greater sample size required for a comparator would not have been feasible from a timing or economic perspective [29]. No attempt was made to evaluate the impact of geographical location or ethnicity. Subtle differences in outcome may exist between the various DOAC and/or be influenced by ethnic or geographic factors, although the pharmacodynamic characteristics of the FXa-inhibitors are rather similar and mainly depend on renal function.

To account for the challenges of data collection in a registry, patients were provided with memory aids to support data recall, data elements were reviewed at the patient level, and all critical events were centrally adjudicated. Offsetting any loss in quality because of missing data or lack of mechanisms to support compliance is the ability of an observational study to accurately reflect current clinical practice without external influence.

## Conclusions

Detailed age-based subset analysis of the original EMIT data shows minimal differences in patient risk factors linked to age; however, management differed in the elderly. The overall rates of MB and CRNMB were low, and there were no clear age-related trends in incidence. However, the incidence of ischemic complications in the elderly trended higher than in the younger population. This difference is better explained by a longer duration of interruption (both pre- and post-procedural) in the elderly and very elderly, rather than differences in procedural risk, thus supporting the view that increasing age should not be a barrier to adequate anticoagulation [30]. Optimal management that balances thromboembolic and bleeding risk in the elderly is yet to be fully defined.

## Supplementary information

**Additional file 1**. EMIT study Ethics Committees.

## Data Availability

The datasets generated and/or analysed during the current study are not publicly available because this study is sponsored by a pharmaceutical company and the raw data are proprietary, but they are available from the corresponding author on reasonable request.

## Abbreviations

ACS: Acute coronary syndrome
AF: Atrial fibrillation
CRNMB: Clinically relevant non-major bleeding
DVT: Deep vein thrombosis
EMIT: Edoxaban management in diagnostic and therapeutic
ISTH: International Society of Thrombosis and Haemostasis
MB: Major bleeding
PE: Pulmonary embolism
SEE: Systemic embolic events
TIA: Transient ischemic attack
