# Correction to: Elderly patients with atrial fibrillation in routine clinical practice: peri-procedural management of edoxaban oral anticoagulation therapy is associated with a low risk of bleeding and thromboembolic complications: a subset analysis of the prospective, observational, multinational EMIT-AF study

**DOI:** 10.1186/s12872-021-01873-2

**Published:** 2021-02-15

**Authors:** M. Unverdorben, C. von Heymann, A. Santamaria, M. Saxena, T. Vanassche, J. Jin, P. Laeis, R. Wilkins, C. Chen, P. Colonna

**Affiliations:** 1 Global Medical Affairs Specialty and Value Products, Daiichi Sankyo Inc., 211 Mt Airy Road, Basking Ridge, NJ 07920 USA; 2 Department of Anaesthesia and Intensive Care Medicine, Emergency Medicine, and Pain Therapy, Vivantes Klinikum Im Friedrichshain, Landsberger Allee 49, 10249 Berlin, Germany; 3 Hematology Department, University Hospital Vilaopó y Torrevieja, Alicante, Spain; 4 William Harvey Research Institute, Barts Health NHS Trust, Charterhouse Square, London, EC1M 6BQ UK; 5 Department of Cardiovascular Sciences, University Hospitals (UZ) Leuven, Leuven, Belgium; 6 Daiichi Sankyo, Medical Affairs Europe, Munich, Germany; 7 QPS Consulting, LLC, 19884 Naples Lakes Terrace, Ashburn, VA 20147 USA; 8 Department of Cardiology, Polyclinic of Bari - Hospital, 70124 Bari, Italy

## Correction to: BMC Cardiovasc Disord (2020) 20:504 10.1186/s12872-020-01766-w

After publication of the original article [1], the authors identified an error in Table 1 related to two values on EHRA bleeding risk-low row.

The correct values are listed below:

**Table:** 

EHRA bleeding risk-low n, (%)	174 (62.1)	580 (49.9)	457 (55.1)	297 (48.5)	283 (51.5)	252 (49.0)	45 (45.9)

The original article has been corrected.
